# Supplementary material for: A Real-Time Detection Device for the Rapid Quantification of Skin Casual Sebum Using the Oil Red O Staining Method
Source: Sensors (Basel). 2022 Apr 14;22(8):3016. doi: 10.3390/s22083016 (PMC9029847; doi:10.3390/s22083016)
Supplement: Supplementary file 1 [file sensors-22-03016-s001.zip › sensors-1667980-supplementary-update.pdf]

Supplementary Materials. The code for color sensor-based device operation.

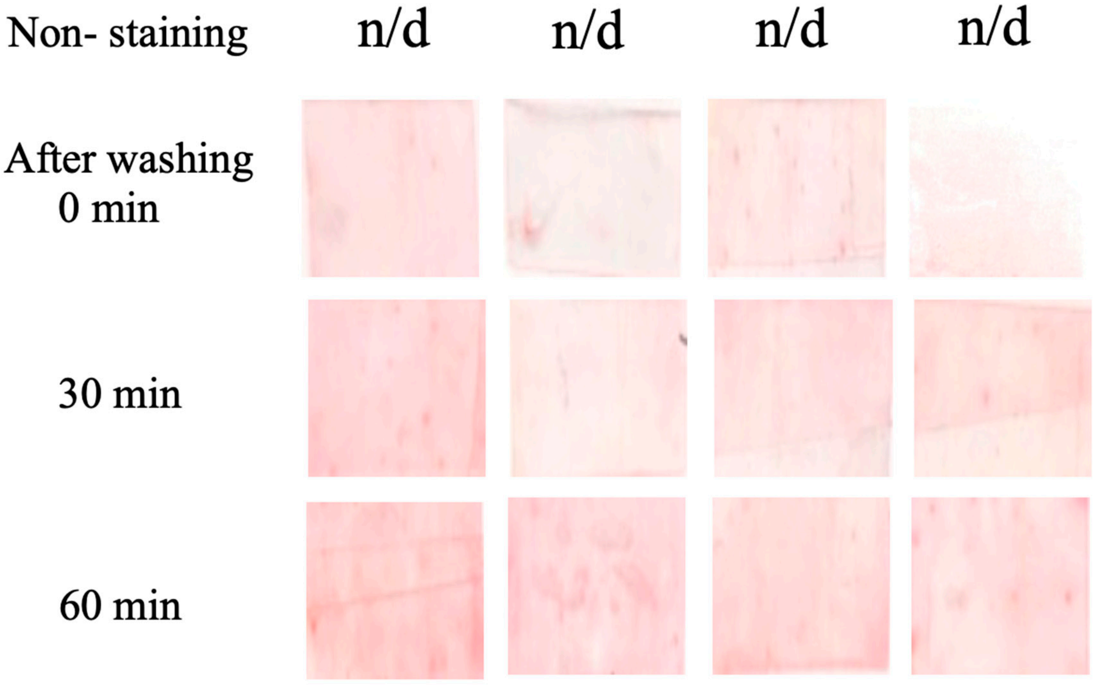

Figure S1. The visual change in color density of stained sebum from the sebum film by time period after facial washing.

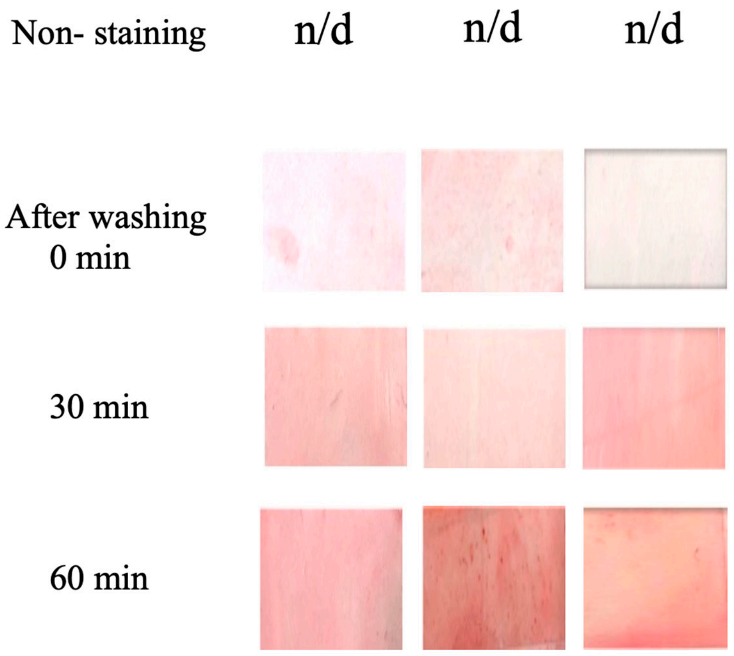

Figure S2. The visual change in color density of stained sebum from the sebum film by time period after facial washing.

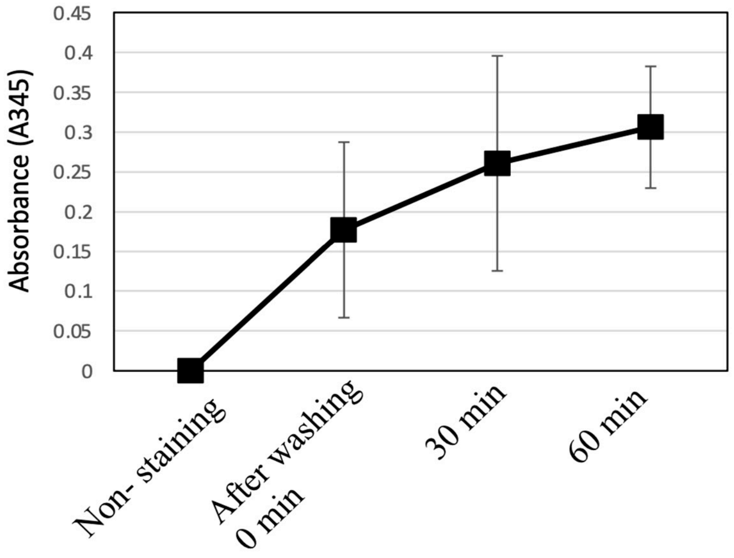

Figure S3. Absorbance measurement result of isolated oil from ORO-stained sebum film by sebum replacement time.

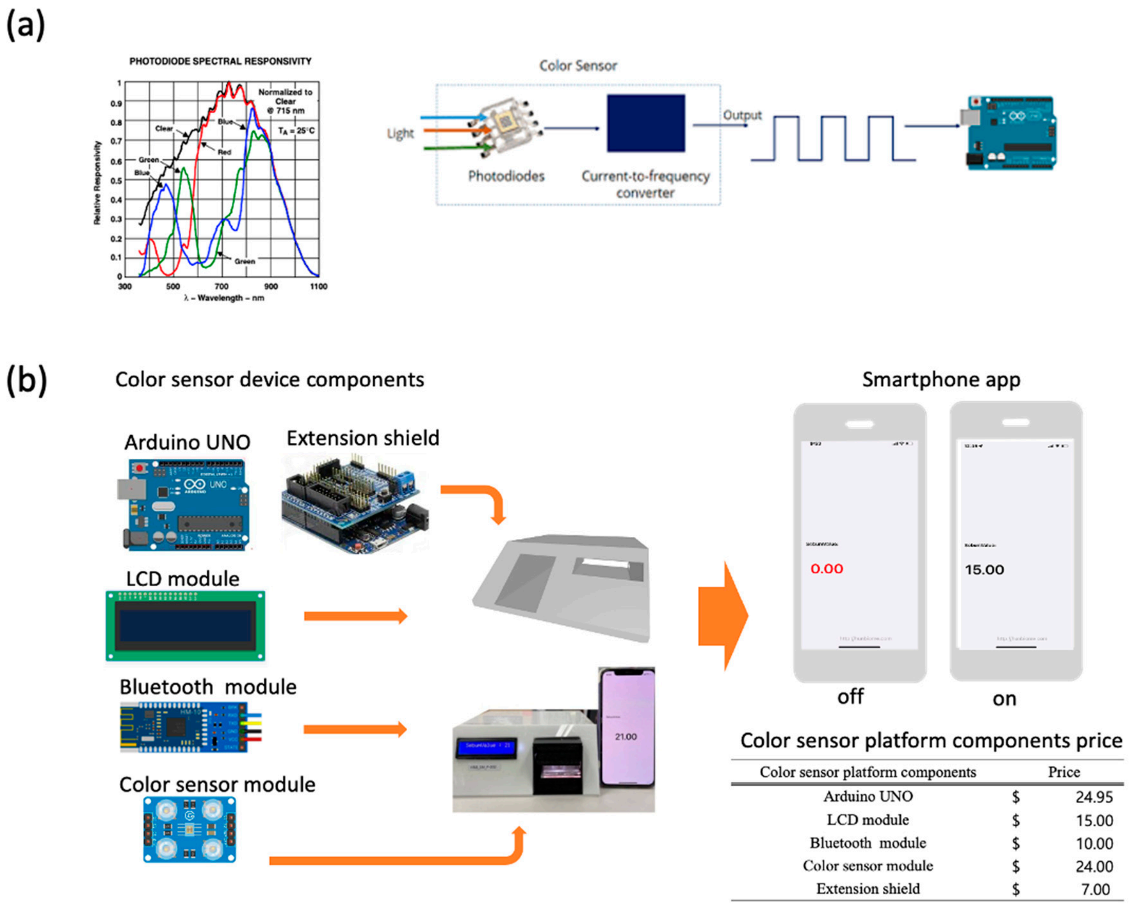

Color sensor platform components	Price	
Arduino UNO	$	24.95
LCD module	$	15.00
Bluetooth module	$	10.00
Color sensor module	$	24.00
Extension shield	$	7.00

Figure S4. Schematic diagram of systems for color detection based on sensor. (a) The wavelength ranges of color detection of TCS3200 color sensor and schematic diagram for color detection. (b) The fabricated sebum measuring system and price of components.

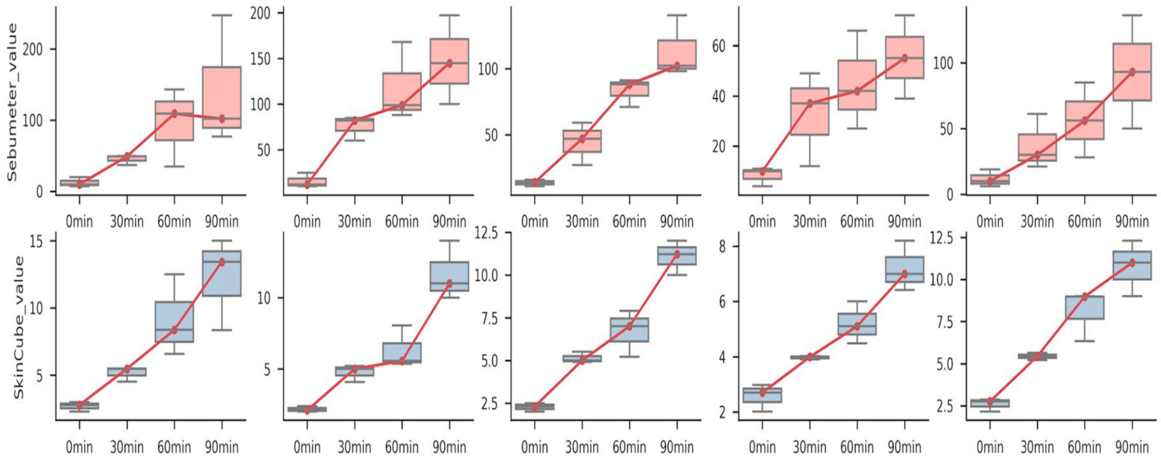

Figure S5. Comparison of ORO-stained color intensity and results of Sebumeter® on sebum replacement time.

Supplementary Table S1. The participants' information for sample collection of facial skin sebum.

Participant	Sex	Age	Skin type	Skin disease
PM1	male	45	oily	N
PM2	male	35	oily	N
PF1	female	29	dry	N
PF2	female	30	oily	N
PM3	male	45	oily	N
PM4	male	35	oily	N
PF3	female	30	oily	N
PS1	male	44	dry	N
PS2	male	38	oily	N
PS3	female	32	oily	N
PS4	female	28	dry	N
PS5	male	46	oily	N

Supplementary Table S2. Comparison of ORO-stained color intensity and results of Sebumeter® on sebum replacement time.

Measuring platform	Sebum replace time	PS1				PS2				PS3				PS4				PS5			
		Intensity	Average	SD	R^2	Intensity	Average	SD	R^2	Intensity	Average	SD	R^2	Intensity	Average	SD	R2	Intensity	Average	SD	R^2
	After washing 0 min	20				12				14				10				10			
		10	12.33	6.09		25	15.67	7.28		16	13.67	2.52		11	8.33	3.39		19	11.67	6.66	
		7				10				11				4				6			
	3 0min	37				60				27				37				21			
		49	45.33	7.23		85	75.67	12.21		59	44.33	16.17		49	32.67	16.88		61	37.33	18.77	
Sebumeter		50			0.99	82			0.98	47			0.998	12			0.97	30			0.98
	60 min	35				99				91				42				28			
		143	95.67	49.39		168	118.33	38.78		88	83.33	10.79		66	45.00	17.60		85	56.33	28.50	
		109				88				71				27				56			
	90 min	77				145				98				55				50			
		247	142.00	82.10		197	147.33	43.42		140	113.33	23.18		72	55.33	14.76		136	93.00	43.00	
		102				100				102				39				93			
	After washin	2.78	2.68	0.33	0.99	2.1	2.17	0.19	0.94	2.5	2.27	0.25	0.94	2.98	2.56	0.45	0.99	2.15	2.59	0.39	0.99
		2.99				2				2				2.01				2.87			

	g<br>0 min	2.28			2.4			2.3			2.7			2.76		
Color sensor device	30 min	4.5			5			5.5			3.9			5.65		
		5.5	5.16	0.51	4.07	4.76	0.54	4.9	5.13	0.32	3.99	3.97	0.06	5.44	5.44	0.22
		5.47			5.2			5			4.02			5.22		
	60 min	6.57			5.56			7			5.11			6.33		
		12.5	9.15	2.72	5.37	6.33	1.34	5.2	6.70	1.37	6.01	5.20	0.68	8.99	8.11	1.54
		8.37			8.05			7.9			4.49			9		
	90 min	8.35			10			10			7			9		
		15	12.26	3.11	14	11.67	1.86	12	11.07	1.01	8.2	7.21	0.81	11	10.77	1.66
		13.44			11			11.22			6.42			12.3		

Supplementary Table S3. Comparison of the conventional measuring methods, including the detection of sebum content and the ORO staining method.

Detection contents	Methods of assement							
	Solvent	Absorbent pad	Bentoite clay	Sebutape	Sebumeter	Lipomenter	Sebufix	ORO-stained methods
Casual sebum level	✓				✓	✓		✓
Sebum excretion rate	✓	✓			✓	✓		✓
Instant sebum delivery				✓			✓	✓
Follicular excretion rate				✓			✓	
Glandular parameter				✓			✓	

Supplementary Materials

```
#include <LiquidCrystal_I2C.h>
#include <SoftwareSerial.h>
#include <Wire.h>

#define S0 4
#define S1 5
#define S2 6
#define S3 7
#define sensorOut 8
```

```
LiquidCrystal_I2C lcd (0x27, 20, 4);
SoftwareSerial BTSerial(3,2);

// Stores frequency read by the photodiodes
const int numReadings = 10;

double redFrequency = 0;
double greenFrequency = 0;
double blueFrequency = 0;

// Stores the red. green and blue colors
double redColor = 0;
double greenColor = 0;
double blueColor = 0;

//calibration
int redMin = 15;
int redMax = 255;
int greenMin = 16;
int greenMax = 255;
int blueMin = 13;
int blueMax = 255;
  // map(value, fromLow, fromHigh, toLow, toHigh)
  // redColor = map(redFrequency, 70, 120, 255,0);
  // greenColor = map(greenFrequency, 100, 199, 255, 0);
  // blueColor = map(blueFrequency, 38, 84, 255, 0);

// sebum value calculation

double SebumValue = 0;
int repeat = 200;

void setup()
 {
```

```
  // Begins serial communication
  lcd.begin(16, 2);
  Serial.begin(9600);
  BTSerial.begin(9600);

  pinMode(S0, OUTPUT);
  pinMode(S1, OUTPUT);
  pinMode(S2, OUTPUT);
  pinMode(S3, OUTPUT);

  // Setting the sensorOut as an input
  pinMode(sensorOut, INPUT);

  // Setting frequency scaling to 20%
  digitalWrite(S0,HIGH);
  digitalWrite(S1,LOW);

  lcd.init();
  lcd.backlight();
  lcd.setCursor(0,0);
  lcd.print("Hello HuNBiome");
  lcd.setCursor (0,1);
  lcd.print("I'm sebum reader");

  Serial.println("Hello HuNBiome");
  Serial.println("I'm sebum reader");

  delay(3000);

//lcd connection
  lcd.clear();
  Serial.println("Cleared!");
  double sumSV = 0;
  double meanSV = 0;
```

```
for (int i=0; i<=repeat; i=i+1)
{

// Setting RED (R) filtered photodiodes to be read
digitalWrite(S2,LOW);
digitalWrite(S3,LOW);

// Reading the output frequency
redFrequency = pulseIn(sensorOut, LOW);
redColor = map(redFrequency, redMin, redMax, 255, 0);

// Setting GREEN (G) filtered photodiodes to be read
digitalWrite(S2,HIGH);
digitalWrite(S3,HIGH);

// Reading the output frequency
greenFrequency = pulseIn(sensorOut, LOW);
greenColor = map(greenFrequency, greenMin, greenMax, 255, 0);

// Setting BLUE (B) filtered photodiodes to be read
digitalWrite(S2,LOW);
digitalWrite(S3,HIGH);

// Reading the output frequency
blueFrequency = pulseIn(sensorOut, LOW);
blueColor = map(blueFrequency, blueMin, blueMax, 255, 0);

SebumValue = redColor-(greenColor+blueColor)/2;
sumSV = sumSV + SebumValue;
Serial.println(i);
Serial.println(SebumValue);
Serial.println();

}
meanSV = sumSV / repeat;
```

```
  Serial.println("sumSV");
  Serial.println(sumSV);
  Serial.println("meanSV");
  Serial.println(meanSV);
  Serial.println();
  Serial.println();

// LCD
  lcd.init();
  lcd.backlight();
  lcd.setCursor(0,0);
  lcd.print("Hello HuNBiome");
  lcd.setCursor(0,1);
  lcd.print("I'm sebum reader");

  Serial.println("Hello HuNBiome");
  Serial.println("I'm sebum reader");

  // Bluetooth
  if (BTSerial.available()) {
    Serial.println(SebumValue);
  }
    lcd.clear();
    lcd.setCursor(0,0);
    lcd.print(" Sebum : ");
    lcd.print(meanSV, 2);
    lcd.setCursor(0,1);
    lcd.print(" Reset for new");
}

void loop() {
}
```
